# Development of Cracking Patterns in Modified Cement Matrix with Microsilica

**DOI:** 10.3390/ma11101928

**Published:** 2018-10-10

**Authors:** Maciej Szeląg

**Affiliations:** Faculty of Civil Engineering and Architecture, Lublin University of Technology, 40 Nadbystrzycka Str., 20-618 Lublin, Poland; maciej.szelag@pollub.pl; Tel.: +48-81-538-4428

**Keywords:** cracking patterns, image analysis, cement matrix, microsilica, dispersion system

## Abstract

The paper evaluates the cracking patterns created on the surface of a microsilica-modified cement matrix, which has been subjected to exposure at elevated temperatures. To do this, image analysis techniques were used, and the structure of the cracks was described by the stereological parameters. Four series of specimens were tested and in two of them, microsilica was used as a 10% replacement for the cement content. Using the theory of dispersion systems, the factors affecting the cracks’ characteristics were identified. Additionally, the development process of cracking patterns due to the thermal interaction was schematically modeled. In addition, the analysis of the local microstructure of the cement matrix was performed by means of a scanning electron microscope and energy dispersive x-ray spectroscopy.

## 1. Introduction

**Figure 1:**
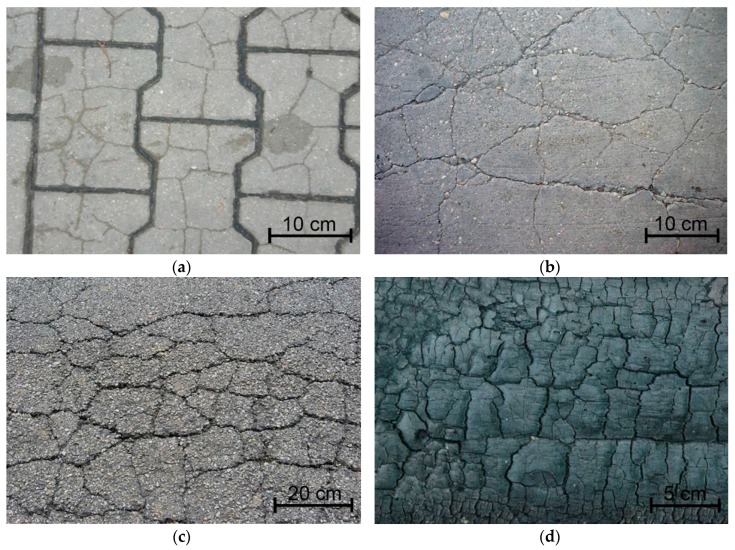
Cracking patterns visible on different materials: (**a**) concrete pavement stone; (**b**) concrete floor [19]; (**c**) asphalt surface [20]; (**d**) burnt wood [21].

The changing nature of thermal interactions in which cement composites operate results in a number of phenomena in the material’s structure, the effect of which are volumetric deformations, which can cause composite cracking [1,2,3]. Propagating cracks combine or intersect to form a cracking pattern also known as thermal cracks, cluster cracks, or map-cracking [4,5,6]. These characteristic cracking patterns are crucial to almost all building materials that have been subjected to environmental (external) influences, examples of which are shown in Figure 1.

In cementitious materials, the microsilica (MS) is mainly used to improve their performance. Its use significantly increases the tightness of composite (greater resistance to chloride ion penetration, sulfate corrosion, etc.), increases the early strength, and strengthens the weakest place in concrete—the interfacial transition zone (ITZ) by reducing its local porosity and reacting with Ca(OH)_2_ [7,8,9,10]. MS is also an indispensable component of high and ultra-high strength concretes [11]. However, the presence of MS increases the risk of more cracks creation in the early maturation phase due to the occurrence of a larger shrinkage, by about 15%, compared to concrete that does not have this addition [1]. Concrete containing 10% of MS has better thermal resistance (lower porosity, higher compressive strength, and higher bulk density) at temperatures of 450 °C and 600 °C, compared to classical concrete [12]. Saad et al. [13] used MS as a 10% substitute of cement mass. This resulted in an increase of 64.6% in compressive strength after exposure to 600 °C. The use of MS as a cement substitute in the amount of 20% and 30% increased the strength by 28%, at the temperature as above.

The behavior of the cement matrix, at elevated temperatures, is complex, due to its chemical diversity and the presence of multiple levels of structural heterogeneity [4]. The microstructure and chemical composition of the hardened cement matrix changes continuously and gradually as temperatures increase from room temperature, up to 1000 °C [14,15,16]. Degradation of the material depends significantly on the temperature to which it is exposed, but heating kinetics also have a significant influence. The sudden heat load increases the probability of creating defects (i.e., cracks) [1,17,18].

Studies [15,16,22,23] have been carried out about the performance of the cement matrix subjected to elevated temperatures. However, most often these considerations apply only to the evaluation of the mechanical and physical parameters. The cracking patterns have rarely been characterized so far because it is challenging to visually examine the cracking process at elevated temperatures, and most reports of thermal cracking have been based on numerical analyses [3,24,25]. The structure of thermal cracks in cementitious materials was the subject of research conducted by Xu et al. [26], who performed the crack density evaluation. The crack density (the term proposed by Mobhaser et al. [27]) was defined as the ratio of the cumulative crack length to the crack area under evaluation. The cracking pattern was observed for concretes and cement pastes after exposure to several temperatures, from 250 °C to 650 °C. After the influence of 250 °C, it was noticed that there was a much greater decrease in tensile strength than compressive strength, which may be related to the orientation of cracks on the sample’s surface. The rapid chloride diffusion test indicated a greater deterioration in composites durability when the crack density was greater. However, the studies conducted by Kim et al. [28] proved that spalling, which is one of the most damaging effects of fire on cementitious materials, was largely caused by a created network of cracks.

The cracking patterns were the subject of analysis, also when the factor causing its formation was drying shrinkage. Wang et al. [29] observed such cracking structures on the surface of modified cement matrix with various types of fibers. Total cracks area was measured and it was noticed that it was reduced by 30–40% compared to classical cement paste. It was found that the reason for this was the appearance of an additional group of large pores which might reduce the capillary pressure in the matrix, which affects the reduction of plastic shrinkage and total cracks area. Bakhshi and Mobasher [30] obtained similar results in their research and the image analysis techniques were used to determine the cracks area and crack width.

Bisschop and Wittel [31] studied the unconfined hardened cement matrix that crack upon drying. Using image analysis crack, patterns on the drying surface was evaluated. It was noticed that such parameters as the sample’s thickness and drying rate affect the density of cracking pattern as well as a crack’s depth. This knowledge is helpful to predict the degree of microcracking of drying concrete, surfaces such as in bridge decks and pavements.

In a liquid phase, cement paste can be considered as a highly concentrated dispersion system, which is a dense suspension of cement grains in water. The structure of this suspension depends mainly on *w*/*c* (water/cement) ratio [32], the degree of cement grinding, and the forces between the grains and the water molecules [33,34]. The intermolecular forces depend on the surface charge of the grains, concentration of ions in a solution, and adsorption. All particles immersed in water have a surface charge, which arises as a result of ionization of surface groups, hydration, dissociation, and ion exchange between phases [35,36]. Cement grains are polimineral, so different surface charges may occur on their surface. Thus, the cement grains flocculate in suspensions to form structural aggregates, which in the static state, tend to merge into one continuous structure [37].

Costa and Massazza [38] distinguished four basic types of interactions between the solid and liquid phases, depending on the size of the particles (the approximate grain size is given after the dash):

(1) attractive capillary forces—from 1 to 0.1 mm;
(2) attractive van der Waals’ forces—from 0.1 to 10^−4^ mm;
(3) repulsive Coulomb’s forces—from 10^−2^ to 10^−6^ mm;
(4) repulsing pressure—from 10^−2^ to 10^−6^ mm.

In the cement matrix, where the proportion of solid phase is significant, capillary forces, van der Waals, and electrostatic interactions play the most important role. The first ones dominate because of their occurrence in size range which is equal to the size of the cement grains. The electrostatic interaction is associated with the formation of a double electric layer around the particle, in a solution, which is not the subject of analysis in this paper, but is discussed in more detail in [34,35,39,40,41].

Between the dispersed phase (cement grains) and the continuous phase (aqueous environment), there is a separation boundary [35,42,43]. Usually, the dispersed phase is characterized by thermodynamic instability, but in a certain period of time, it can be metastable (such a feature is exhibited by cement in a short time after being combined with water). At the initial stage, the solid phase is not transformed, mainly due to the protective layer around the particle, which can be described in a molecular and thermodynamic way [44].

For the molecular description, the van der Waals’s forces, Coulomb’s forces, and hydrogen bonds are used. In the thermodynamic description of the separation boundary, the concept of the surface formation enthalpy is applied [44] as below.

(1) $$\Delta H^{A}=\Delta A\left[\sigma -T{\left(\frac{\partial \sigma }{\partial T}\right)}_{A}\right],$$

where: $\Delta H^{A}$—surface formation enthalpy,

σ—surface tension [N/m],

A—interfacial surface area [m^2^],

T—temperature [K],

$\sigma \Delta A=\Delta G^{A}$—the Gibbs’ free energy.

The basis for the description of this particle aggregation process in the cement-paste dispersion systems is through the use of the DLVO theory [17,35,45,46,47] (from names Derjaguin-Landau-Verwey-Overbeek), which describes the process of forming a double electric layer around the particle, in a solution. This theory defines the total potential energy of interaction between two molecules with a spherical shape, which is a result of the van der Waals’ forces of attraction and repulsive forces (electric forces of the double layer, solvation, and steric effects). However, in the presence of water, capillary forces appear between the solid phase particles, which can be quantitatively calculated [4,6,48] as:

(2) $$F_{K}=\Delta PS_{o}\sigma l,$$

where: $F_{K}$—capillary force,

$S_{o}$—wetting surface,

l—wetting parameter,

σ—surface tension on the phases boundary,

ΔP—change in fluid pressure (the Laplace’s pressure).

The capillary forces are one of the causes of flocculation of cement grains. In dispersion systems involving materials that have binding properties, capillary forces largely shape the physical and mechanical properties of a composite [4,43]. In an aquatic environment, capillary forces produce mutual attractions between the particles. A result of this process is a creation of the binding units. Depending on the size of the particles, binding units of different sizes are formed, which can be defined as the clusters [4,43,49,50,51]. The cluster-forming process in cement paste has a fractal character, which results in clusters occurring at all levels of material heterogeneity. If an external load is applied, the shape and size of the clusters correspond with properties of the resulting cracking patterns [42,52,53].

In this study, the author investigated the cracking patterns, which were created as a result of thermal shock on the MS-modified cement paste. The cracks were quantitatively described using stereological parameters, obtained from the image analysis of the surface. In addition, an analysis of the microstructure with the use of the scanning electron microscope (SEM) and the energy dispersive x-ray spectroscopy (EDS) was performed. It was attempted to identify factors, which influence the properties of the cracking patterns (their size, width, etc.). Additionally, a scheme for the process of development of cracking patterns was proposed, which is the scientific novelty of this work. For this purpose, the cement matrix was considered to be a highly-concentrated dispersion system. The results of these studies, in addition to the importance of the idea, can also be useful in practice. Knowledge of these dependencies will help to control the technological process in terms of the selection of appropriate components of cement composites and their mutual proportions, in order to reduce the cracking process.

## 2. Materials and Methods

### 2.1. Components Used and Sample Preparation

Four series of cement pastes were tested. Three different *w*/*b* (water/binder) ratios equal to 0.4, 0.5, and 0.6 were evaluated. The binder is considered to be the total content of cement and the MS in the specimen. The first series was a pure cement matrix made of CEM I 42.5R ordinary Portland cement (Cemex, Chelm, Poland). In the second one, the same cement was used but 10% of its mass was replaced by MS. The third and the fourth series were analogous to the first and second ones, with the difference being that CEM I 52.5R ordinary Portland cement was used, instead.

The types of cement used had very similar chemical compositions; as was determined by energy dispersive X-ray fluorescence, by means of a spectrometer Epsilon 3 (Panalytical, Almelo, Netherlands). However, they differed in the specific surface area—as shown in Table 1. A similar oxide composition of the cement type, translated directly to a similar mineral composition (calculated using the Bogue’s equations [54]). The different series were designed to determine the impact of MS on cracking patterns, as well as the effect of the concentration of the dispersed phase (variable *w*/*b*) and the effect of the grain size of the dispersed phase, while minimizing the effect of chemical diversity of the cement type.

All samples had dimensions of 40 × 40 × 160 mm^3^. Cement paste was molded in two layers, compacted one after the other using a standard shaker, according to EN 196-1 [55]. After 24 h, the samples were unmolded and then they were stored for 28 days in dry air conditions, with an average relative humidity of 50% and an average temperature of 22 °C.

### 2.2. Elevated Temperature Loading

After 28 days of maturation, the samples were subjected to a thermal shock. First, a furnace was preheated to a temperature of 250 °C. Then samples were kept in it, for 4 hours. After heating, samples were taken out from the furnace and cooling took place through the natural temperature drop in a laboratory (about 20 °C). The use of such a temperature-loading method was determined by enforcing the creation of cracking patterns, as a result of the volumetric deformations—the material’s thermal expansion in the heating phase and shrinkage in the cooling phase. At the tested temperature range, mainly free water evaporated and cement hydration products still exhibited a high chemical stability [14]. The steam pressure created in the pores and capillaries of a cement matrix caused cracks propagation, and transformation of microcracks into macrocracks, which were visible on the surface of the specimen. In this way, the cracking pattern was extracted. The temperature load parameters were selected on the basis of tests carried out at different temperatures and at different load duration, within the framework of the previous author’s studies [6,48,56,57,58,59].

### 2.3. Basic Physico-Mechanical Characteristics of the Cement Pastes

Table 2 summarized the values of mechanical properties and bulk density of the modified cement pastes. All the tests were performed in accordance with the following standards:

*f_c_*—the compressive strength—EN 12390-3 [60],
*f_cf_*—the three-point bending flexural strength test—EN 12390-5 [61],
*D*—the bulk density—EN 12390-7 [62].

The index (*R*) represented the reference value, which was obtained after twenty-eight days of maturation. The index (*T*) represented the value obtained after an elevated temperature load and after cooling down to the room temperature. The *f_c_* results were arithmetic means of twelve samples; *f_cf_* and *D* results were arithmetic means of six samples. Values of the coefficients of variation are shown in Table 3.

### 2.4. Image Analysis

In order to obtain cracking patterns for the analysis, specimens were scanned using the optical scanner—Epson Perfection V33 (Seiko Epson Corporation, Suwa, Japan), which had a maximum scanning resolution equal to 4800 DPI. The scanning was performed with the resolution of 2400 DPI, in an eight-bit grayscale, and with the unsharp mask set to a high level. Scanning in such a high resolution was conditioned to obtain the most detailed image of the sample’s surface. The 1 mm of scanned area accounted for 94.5 pixels, which allowed for positive crack identification, whose thickness was equal to 2–3 pixels (width of about 0.021 mm). Detection of the cracks with such a width should be considered to be a good result, especially when magnifying optics was not used, and cracks with such width were not visible to the naked eye.

For the quantitative description of cracking patterns, three stereological parameters were proposed to be measured, the cluster average area ($\bar{A})$, the cluster average perimeter ($\bar{L})$, and the crack average width ($\bar{I})$. A cluster in this sense means an area on the sample’s surface, which is limited on each side by a crack, or is limited by a crack and the sample’s edge.

**Figure 2:**
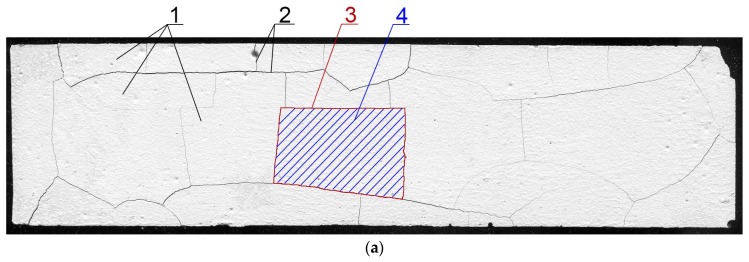

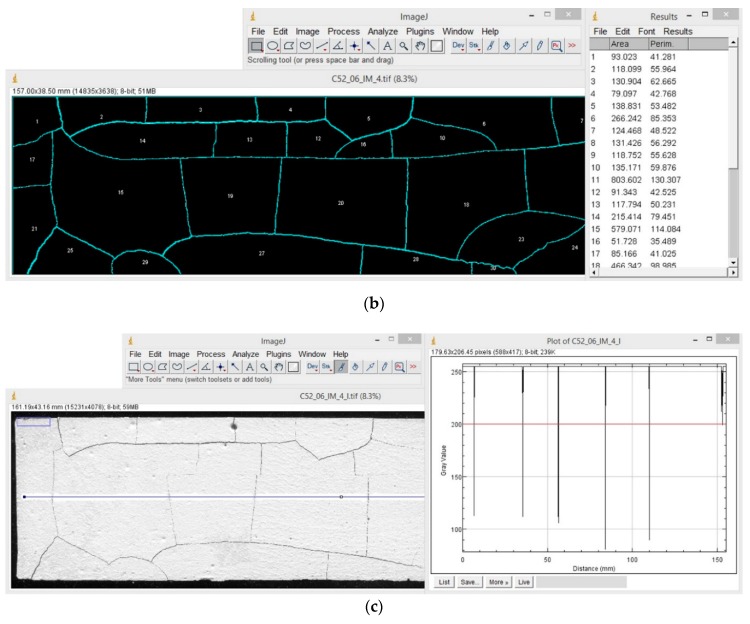
The image of a scanned surface of the cement paste [59]: (**a**) Image obtained after scanning; (**b**) processed and measured ($\bar{A}$ and $\bar{L}$) image; (**c**) processed and measured ($\bar{I}$) image; 1—clusters; 2—cracks forming a cracking pattern; 3—cluster perimeter; 4—cluster area.

Processing and image analysis was performed using ImageJ software (v.1.51j8, National Institute of Health, Bethesda, Rockville, MD, USA). The measurement was limited to an area of 157 mm × 38.5 mm. This was due to the necessity of maintaining a constant surface area for each specimen as the cement matrix deformed under the influence of the temperature gradient. The images were processed using treatments, such as contrasting, sharpening, and thresholding. Next, by using the “analyze particles” tool the $\bar{A}$ and $\bar{L}$ were measured. To measure the $\bar{I}$ the “plot profile” module was used. The measurement procedure has been described in detail in Szeląg’s monography [63]. A sample image of a specimen before and after processing is shown in Figure 2.

### 2.5. Local Microstructure Examination

The analysis of the local microstructure of the MS-modified cement pastes was made based on the SEM images (FEI, Hillsboro, OR, USA). The investigation was conducted on the samples subjected to the thermal load, after cooling the samples to the room temperature. The samples were broken off from the inner part of a specimen. Next, they were dried in 105 °C, for 24 h. In order to obtain a conductive layer, samples were sputtered with carbon. On the same samples, the EDS (FEI, Hillsboro, OR, USA) analysis was carried out, which allowed for the identification of the local chemical composition.

## 3. Results and Discussion

### 3.1. Properties of the Cracking Patterns

**Figure 3:**
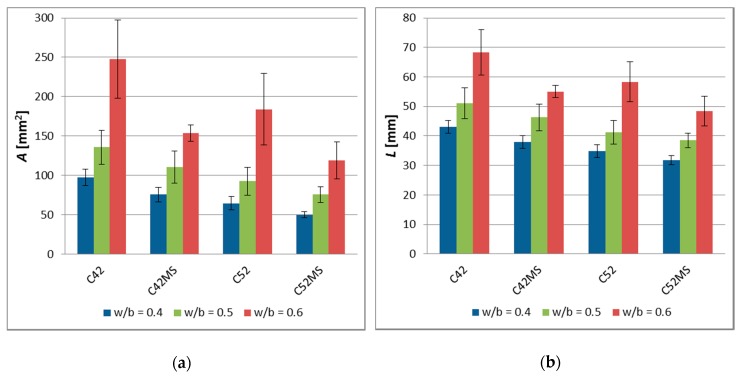

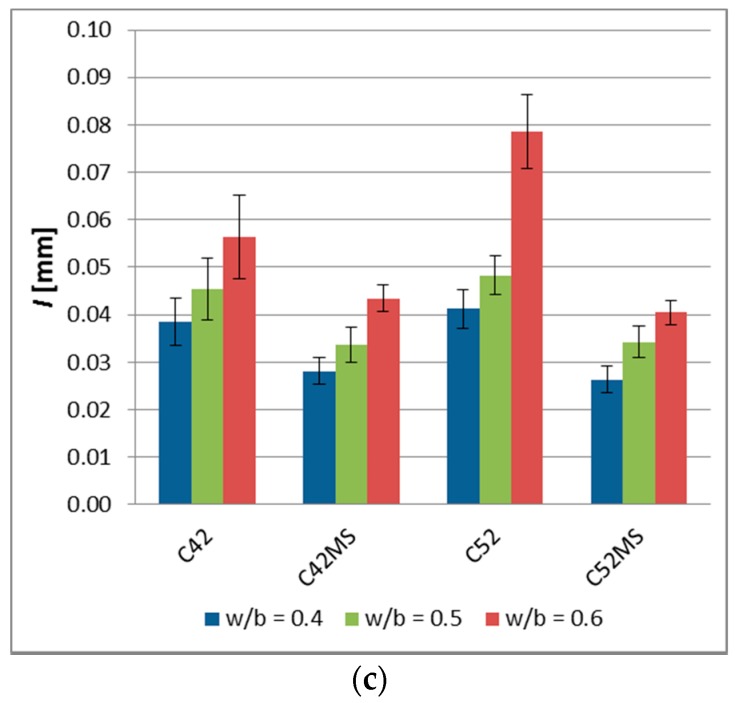
The image analysis results of the cement pastes (error bars mean standard deviations): (**a**) Cluster average area—$\bar{A}$; (**b**) cluster average perimeter—$\bar{L}$; (**c**) crack average width—$\bar{I}$.

The values of $\bar{A}$, $\bar{L}$, and $\bar{I}$, and the standard deviation is shown in Figure 3. The results of the image analysis showed that the characteristics of the cracking patterns depend on technological variables in the production process of cement matrix. The cement pastes made with CEM I 52.5R (C52, C52MS) had smaller $\bar{A}$, $\bar{L}$, and $\bar{I}$ values than those made with CEM I 42.5R (C42, C42MS). This meant that the use of cement with greater fragmentation resulted in the formation of a denser network of cracks, on the sample’s surfaces. However, with an increased number of cracks, the crack average width decreased, and for all the series, values of the stereological parameters increased with *w*/*b*.

The use of MS (C42MS, C52MS) reduced $\bar{A}$ in relation to C42 and C52 by an average of 26.3% and 25.4%, respectively, for the samples made with CEM I 42.5R and CEM I 52.5R. Accordingly, the cement’s class did not affect a relative level of reduction in a cluster average area, which resulted from the use of MS. C52MS samples had reached smaller $\bar{L}$ values by an average of 13.6%, than the C52, and C42MS samples which were 10.9% greater than C42. Especially the beneficial effect of the MS present in the cement paste was noted for $\bar{I}$; for cement paste made with CEM I 52.5 there had been a decrease in $\bar{I}$, by an average of 37.9%. For samples made with CEM I 42.5R, a decrease in $\bar{I}$ was by an average of 25.3%.

With the increase in the *w*/*b*, and thus simultaneously with the reduction of the degree of concentration of the dispersed phase, all the studied stereological parameters had increased in value. This meant that the clusters were larger and the crack width increased between them. Cement pastes with *w*/*b* = 0.5 were characterized by a higher $\bar{A}$, $\bar{L}$, and $\bar{I}$ values, respectively, by 45.1%, 20.0%, and 21.0%, in comparison to the matrix with *w*/*b* = 0.4. In the case of the cement pastes with *w*/*b* = 0.6, the values of the above parameters were higher by 144.8%, 55.9%, and 61.2%, respectively.

In his previous work, the author investigated the effect of metakaolinite on the cracking patterns of cement pastes [59]. It was determined that the presence of metakaolinite in the structure of cementitious material increases $\bar{A}$ and $\bar{L}$ (inversely to the microsilica), whereas the influence on $\bar{I}$ depends on the class of cement used.

**Figure 4:**
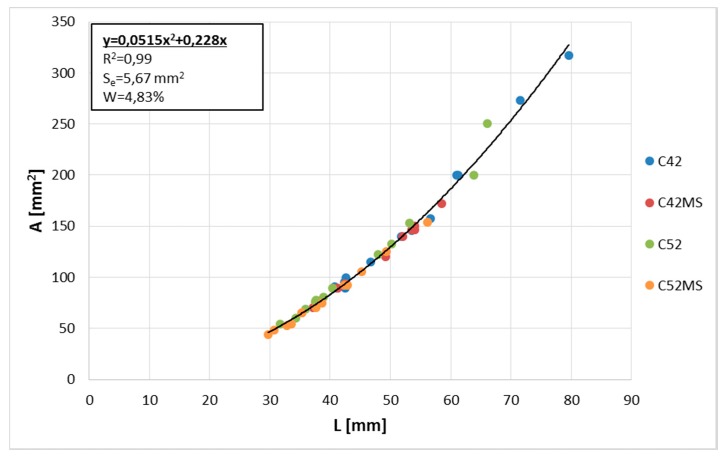
Cluster average area ($\bar{A}$) as a function of cluster average perimeter ($\bar{L}$).

The correlation coefficient (*ρ*) between $\bar{A}$ and $\bar{L}$ was very high, equal to 0.99. Therefore, a geometric relationship of clusters was constant regardless of the technological properties of the material. The global equation of the curve describing $\bar{A}\left(\bar{L}\right)$ relationship was calculated using the least squares method (LSM). This, along with the values of the diagnostic statistics are shown in Figure 4. The *R*^2^ (the coefficient of determination) value of the polynomial showed a value close to one, which indicated a very good fit of the curve to the empirical data. Very low values of *S_e_* (the standard error of estimate) and *W* (the coefficient of random variation) confirmed this fact.

**Figure 5:**
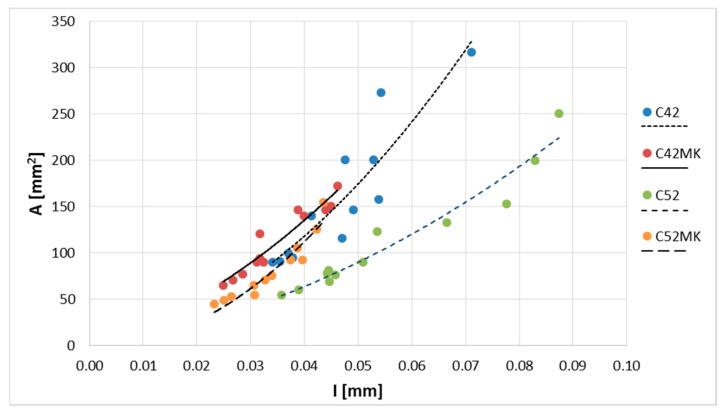
Cluster average area ($\bar{A}$) as a function of the crack average width ($\bar{I}$).

It has been shown above that there was a close relationship between $\bar{A}$ and $\bar{L}$. Therefore, a relation between $\bar{I}$ and other stereological parameters have been limited to describe the relationship with $\bar{A}$ only (Figure 5). The correlation coefficient between $\bar{A}$ and $\bar{I}$ was equal to 0.75. It was found that the relationships could be described with much larger accuracy when the results were presented according to the series. For each correlation coefficient (all greater than 0.9), equations of the curves describing $\bar{A}\left(\bar{I}\right)$ relationship, and values of the diagnostic statistics (Table 4) were calculated. The best fit to the empirical data was obtained for the MS series (C42MS, C52MS); high values of *R*^2^, and low values of *S_e_* and *W* were reached, respectively, which were smaller than C42 and C52.

The content of the main cement phases and the products of cement hydration did not affect the cracking patterns properties because the cement types used were very similar, chemically. Samples made with CEM I 52.5R were characterized by the smaller size of clusters compared to the CEM I 42.5R specimens. A factor for this was the degree of cement-grinding (Blaine’s specific surface area of CEM I 52.5R was 14.6% larger than the CEM I 42.5R).

From Equation (2), it appeared that the capillary forces were proportional to the size of particles, which formed the binding aggregate because a wetting surface increased with the particle size. For larger grains (CEM I 42.5R), a greater capillary force caused a stronger attraction of the particles. Therefore, there was a higher possibility of a nucleation of the larger binding unit. Values of the capillary forces, concentration, and the distribution of a dispersed phase at the dispersing agent were the factors that determined the cluster size.

It could be concluded that with increasing cluster size, its internal energy and cohesion also increased. The increase in the value of these two parameters did not only stem from the capillary force, but it was also dependent on the growth of the Gibbs’ free energy, during the creation of a separation boundary between the phases. Referring to Equation (1) it could be seen that the enthalpy of a surface creation increased with the interfacial contact area, which was greater, the larger the grains of the dispersed phase were. At the time of cluster fixation, connection forces of particles inside the binding unit depended on the enthalpy of interfacial surface creation. During hydration, further development of contacts between the hydrates was followed and short range of physical forces between the CSH nanoparticles and the gel were responsible for a high cohesion of the cement-paste structure [36,64,65].

Beyond cohesion of a binding unit itself, connection forces between the clusters should also be taken into account. This is important because the separation surfaces between the clusters are where the cracks emerge. As a result of an external impact (in this case, the impact of thermal shock) on the separation surfaces between the clusters, cracks began to arise and develop. Taking into account a fractal nature of the process, the cracks created cracking patterns, which were observed at each level of the material structure; starting from a microscale corresponding to the individual grains, ending with a macroscale corresponding to the dimensions of the entire sample.

**Figure 6:**
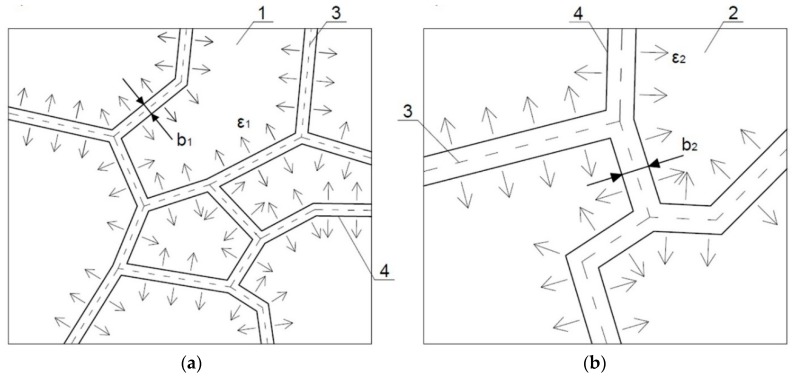
Crack width after the thermal load for clusters of: (**a**) small sizes; (**b**) large sizes; 1, 2—clusters with *A*_1_ and *A*_2_ areas, where *A*_1_ < *A*_2_; 3—separation surface between clusters; 4—edges of a crack after deformation of clusters; *b*_1_, *b*_2_—crack width, where *b*_1_ < *b*_2_; *ε*_1_, *ε*_2_—direction and size of clusters deformations, where *ε*_1_ < *ε*_2_.

In all these series it was noted that as clusters size increased, crack width between them also increased. This was due to the fact that volumetric deformations of individual clusters were dependent on their size, and they were greater, the bigger cluster was. It can be noticed that as a result of breaking contacts between the CSH particles of two adjacent clusters, they were moving away from each other. The increase in the crack width would be greater for larger volumetric deformations of clusters, which is schematically shown in Figure 6. The connection forces between the clusters decreased as the distance between them increased and with the growth of the cluster size. The result was a drop of material cohesion as a whole; the sample was destroyed with less and less load; the lowest strengths obtained samples with the highest *w*/*b*, and therefore with the largest sizes of clusters and the largest crack widths.

It was noted that $\bar{A}$, $\bar{L}$, and $\bar{I}$ increased with *w*/*b*. This was accompanied by an increase in the dispersion degree of cement, which resulted in a more even distribution of capillary forces and intermolecular interactions between grains. At the initial stage of a structure’s self-assembly, grains move at greater distances to create a binding unit, than it would have been in the case of a higher concentration of dispersed phase. Thus, distances between the clusters’ centers increase and in a separation surface between clusters, contacts with reduced strength are shaped. Such conditions cause the contacts between clusters to be broken, at a lower value of an external load. However, the distance between the cement grains begins to decrease in a situation when *w*/*b* decreases. In this case, cement particles were so close to each other that hydrates that had formed on the surface of a cement particle had only to grow a very short distance before reaching the hydrates growing on the surface of the adjacent cement particles. The proximity of the cement particles resulted in the rapid development of very strong bonds and very low porosity, which has been confirmed by other researchers [66,67]. This translated directly to reducing the degree of cracking of the material surface.

Cement pastes modified by MS (C42MS, C52MS) reached lower $\bar{A}$, $\bar{L}$, and $\bar{I}$ values, as compared to the C42 and C52 samples. The use of the MS resulted in a significant reduction in the porosity of the cement paste and modified its properties by binding calcium hydroxide. As a result of this process, hydrated calcium silicates with high strength and durability were created. Creation of secondary CSH phase, additionally, tied the material structure; contacts between the particles could obtain larger sizes and larger local strength. This translated into an increase in energy that needed to be delivered to the sample, such that the resulting stresses led to the disruption of contacts. A physico-chemical strengthening of material increased its cohesiveness; reduced porosity and additional CSH did not allow for the formation of cluster structures of large sizes and with large crack widths. Similar observations have been made by Xu et al. [26]. They noticed that the level of surface cracking was smaller in concrete with other added pozzolana—fly ash, after a thermal interaction.

### 3.2. Cracking Patterns Development Process

**Figure 7:**
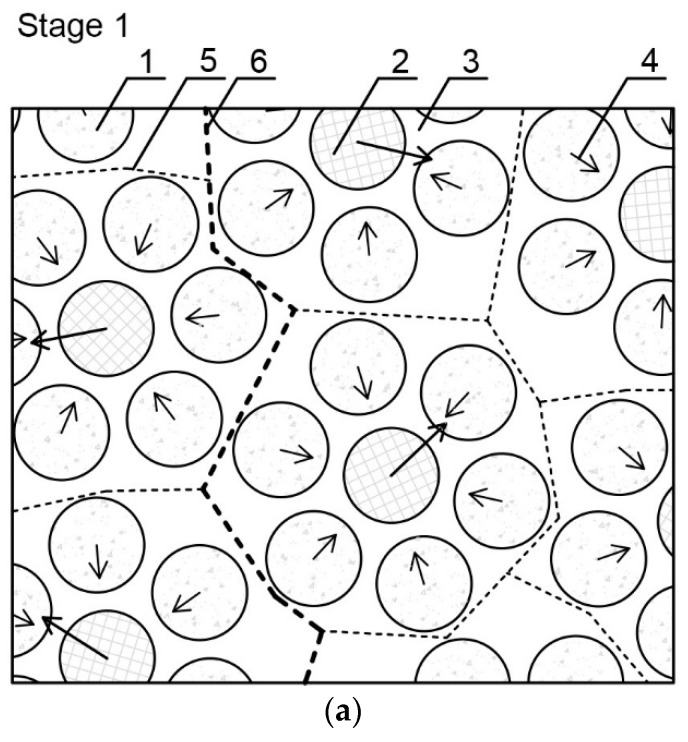

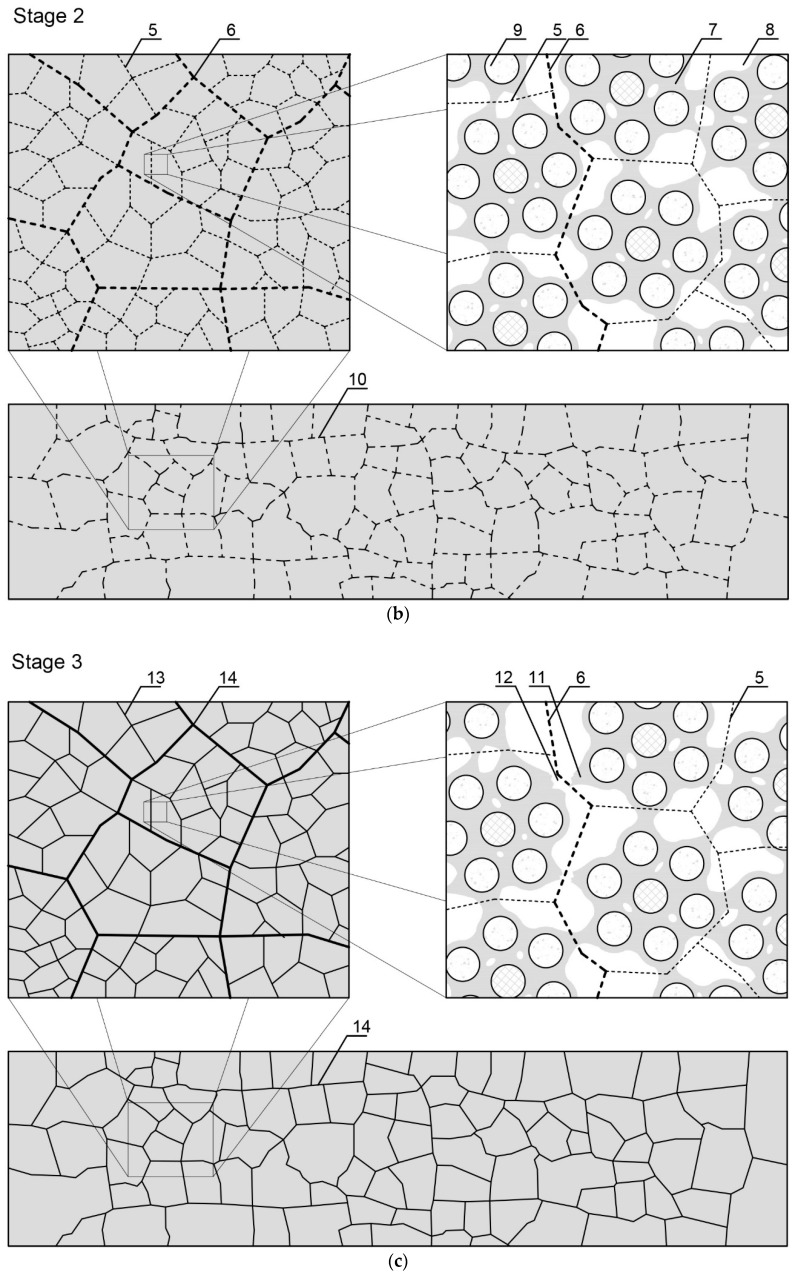
Scheme of the evolution of cluster structures into a cracking pattern as a result of a thermal load: (**a**) stage 1—initial structure organization immediately after cement and water connection; (**b**) stage 2—hydration and formation of a solid structure; and (**c**) stage 3—appearance of stress/strain in the material and formation of a cracking pattern. 1—Surrounding cement particle; 2—forming cement particle; 3—water environment; 4—resultant forces of interaction between cement grains; 5—lower-level separation surface between clusters; 6—higher-level separation surface between clusters; 7—cement hydration products; 8—pores; 9—partially-hydrated cement grain; 10—separation surface between clusters at the highest level of observation; 11—place of contact breaking; 12—crack formation and propagation zone; 13—cracking pattern on a lower level; and 14—cracking pattern on a higher level.

Based on the physical processes occurring in the cement matrix structure and the results obtained from the tests, the schematic process of the evolution of cluster structures into the cracking patterns, caused by a thermal interaction, was proposed. This is shown in Figure 7 and can be described in three stages, as follows.

Stage 1—the initial structure organization immediately after cement and water connection: In the center of a cluster there is a forming particle, in the direction of which, the surrounding particles move. The mutual attraction of cement grains is a result of intermolecular interactions, mainly capillary forces occurring between the grains. The created binding unit can be considered as a lower-level cluster, on which level individual cement grains can be isolated. Then a separation surface is formed between the binding units.

Stage 2—the hydration and formation of a solid structure: As a function of time, the process of hydration of cement grains occurs, which causes the formation of a mineral structure. Jiang et al. [68] indicated that the hardening is caused by the multiplication of contacts between particles. CSH and nanocrystallites, which are formed during the hydration, dramatically increase the number of links of the system, which is interconnected. The cohesion of the cement matrix is caused by the creation of a dense network of CSH nanoparticles; short range physical forces are responsible for linking them together [36,64,65]. The separation surface between clusters runs through contacts between hydrates, and lower-level clusters form a higher-level cluster, at a higher level of the material structure.

Stage 3—the appearance of stress/strain in the material and formation of a cracking pattern: The separation surfaces between the clusters determine the places with the lowest local cohesion in the structure of a hardened cement matrix. At the moment when stresses caused by, e.g., temperature gradient, shrinkage, pressure, mechanical load, etc. appear in the material’s volume, individual clusters start to deform. The creation of a critical strain value between the clusters causes the contact connections between them to break, which results in a formation of a crack. As a result of subsequent deformations, cracks propagate and connect to each other creating a cracking pattern at individual levels of the structure. The nature of the separation surface between the clusters and its parameters, such as length or shape, depend on the quantitative and qualitative composition of the dispersed phase particles and on the intermolecular forces between them, including adhesion and cohesion forces. The exact determination of places where cracks would be created and propagated in the micro- and macrostructure of a material is associated with the formation of separation surfaces between the clusters, at various levels of a structural heterogeneity [5,43,51].

### 3.3. The Local Microstructure of the Cement Matrix

**Figure 8:**
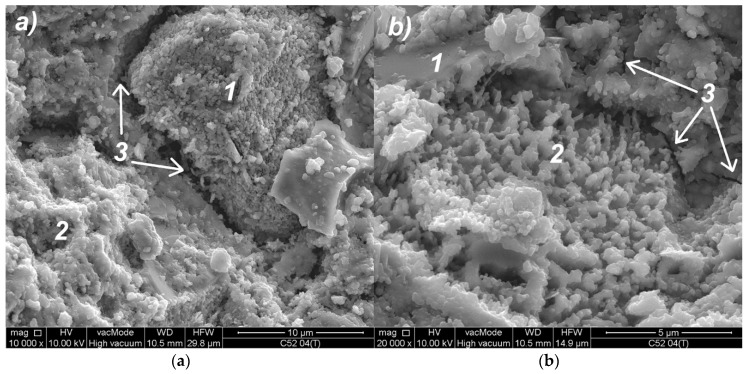
SEM images of the C52 cement paste microstructure: (**a**) Magnification 10,000×; (**b**) magnification 20,000×; descriptions of the indications in the text.

SEM images of modified cement pastes allow an analysis of the material’s morphology and the identification of thermal microcracks. Figure 8a shows the microstructure of the classical cement paste (C52), after the thermal load. Small isometric grains (1) of the CSH correspond to the type III phase of the CSH proposed by Diamond [69]. The CSH is visible in the form of a congested gel (2), which corresponds to the type IV. A crack is visible (3) with bridges formed from the CSH nanoparticles. In Figure 8b, a portlandite plate (1) is observed, which is overgrown with hydrated calcium silicates. In addition, the CSH phase (2) at the borderline of II and III Diamond’s type is visible. In this image, a microcrack (3) passing through the CSH phase is observed. The cracks observed were occurrence zones of the lower-level separation surfaces between the clusters, as compared to the cracking patterns observed in a macroscale. The presence of such cracks at the microstructure level confirmed the fractal character of the cracking patterns.

**Figure 9:**
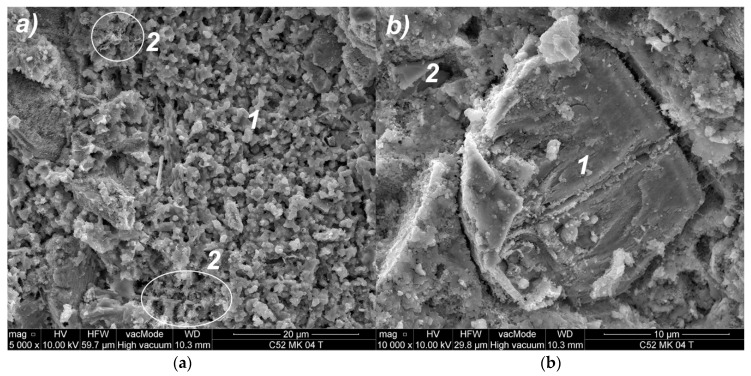
SEM images of C52MS cement-paste microstructure: (**a**) magnification 5000×; (**b**) magnification 10,000×; descriptions of the indications in the text.

As a result of the MS modification, a secondary CSH phase with diversified morphology was formed. Figure 9a shows loosely bound isomorphic crystals of hydrated calcium silicates (1) of III-type, and the CSH II-type of the mesh structure (2). Figure 9b shows a distinct cluster consisting essentially of an amorphous, high-density CSH gel of IV-type (1). Another effect of the use of MS was a quantitative reduction of calcium hydroxide (2); only small crystals were formed, which were incorporated into the CSH phase. In addition, the formation of the secondary CSH phase influenced the reduction of a material’s porosity.

**Figure 10:**
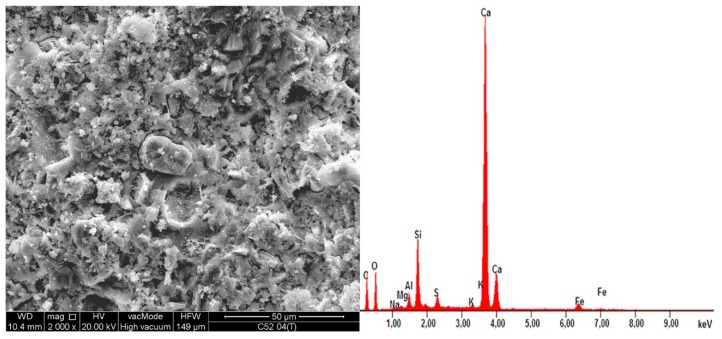
EDS spectra of the C52 sample together with the SEM image of the area analyzed.

**Figure 11:**
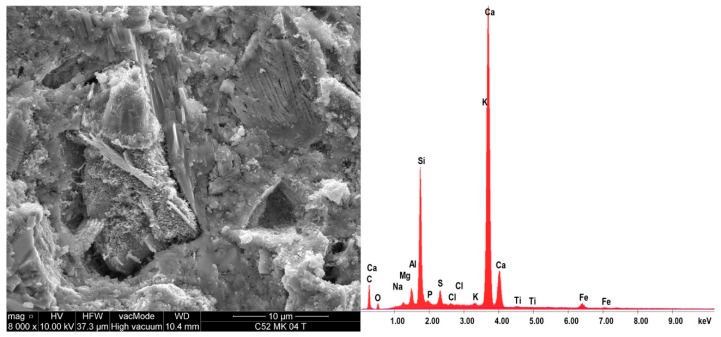
EDS spectra of the C52MS sample together with the SEM image of the area analyzed.

On the same samples, the EDS X-ray microanalysis was performed, results of which are shown in Table 5. The results are presented as percentages of individual oxides in the areas analyzed, which for each sample, together with EDS spectra are shown in Figure 10 and Figure 11. A characteristic for the hardened cementitious materials was the high content of SiO_2_ and CaO; these oxides corresponded to the CSH phase (in the 28 days cement-paste the content of the CSH phase could exceed 70–80%) and the cement hydration products, such as portlandite. The EDS and SEM analysis confirmed that hydrated calcium silicates present in the various morphological forms were the primary constituent of the hardened cement paste. The higher SiO_2_ content was present in the cement paste modified by MS (more by 17.2% compared to C52). This demonstrated the existence of a secondary CSH phase, formed as a result of the MS reaction with Ca(OH)_2_. It is noteworthy that CaO content was only 3.6% lower with respect to C52. The content of Al_2_O_5_ was reduced by 26.3%, which resulted in a slightly different *f_c_* between the samples of C42-C52 and C42MS-C52MS series.

## 4. Conclusions

The paper presents the results of the investigations of cracking patterns of modified cement pastes exposed to elevated temperatures. On the basis of the reviewed literature, and then analyzed and interpreted research results, the following conclusions were drawn:

(1) Computer image analysis tools could be successfully applied to the quantitative description of cracking patterns.
(2) While analyzing the structure of the cement paste as a dispersion system, factors influencing the process of self-assembly of cluster structures were identified at the initial binding stage. Geometric characteristics of the clusters depended on the technological variables in a production process of the cement pastes. The process of a structure self-assembly was shaped by intermolecular interactions in a dispersion medium of the cement paste, and on physico-chemical changes in the system occurring as a result of the cement hydration.
(3) The cluster size was mainly shaped by capillary forces, which increased with the size of cement grains; the larger the size of the cement grains, the larger the size of the clusters.
(4) With a decrease in the amount of binder in the material’s volume (increase in *w*/*b*), the size of clusters and cracks width increased.
(5) The distance between the crack’s edges depended on the clusters’ volume deformations, which increased along with their size.
(6) The three-stage schematic process of the cracking pattern development process was proposed; it described the evolution of the cluster structures into the cracking patterns, due to the thermal interaction.
(7) Adding the MS to the cement matrix caused a physico-chemical sealing of material structure; this reduced the size of clusters and the width of cracks between them.
(8) The microstructure analysis of the modified cement pastes, using SEM and EDS, indicated a presence of the cluster structures at lower levels of the material structure, which confirmed the fractal nature of cracking patterns. The analysis also allowed the identification of structural differences between individual samples of the cementitious material.

## Figures and Tables

**Table 1:** Chemical and mineral components and Blaine’s specific surface area of the ordinary Portland cement used in the study.

**Cement’s Class**	**Chemical Analysis [%]**								
	**SiO_2_**	**Fe_2_O_3_**	**Al_2_O_3_**	**CaO**	**MgO**	**SO_3_**	**Cl**	**Na_2_O**	**K_2_O**
CEM I 42.5R	20.18	3.39	4.38	64.79	1.17	2.91	0.083	0.26	0.49
CEM I 52.5R	20.19	3.30	4.33	64.76	1.17	3.16	0.078	0.26	0.48
**Mineral Composition [%]**					**Blaine Specific Surface Area [cm^2^/g]**				
	**C3S**	**C2S**	**C3A**	**C4AF**					
CEM I 42.5R	63.41	8.92	5.88	10.31	4010				
CEM I 52.5R	62.97	9.28	5.90	10.03	4596				

C3S—alite-tricalcium silicate—3CaO∙SiO_2_; C2S—belite-dicalcium silicate—2CaO∙SiO_2_; C3A—tricalcium aluminate—3CaO∙Al_2_O_3_; C4AF—brownmillerite—4CaO∙Al_2_O_3_∙Fe_2_O_3_.

**Table 2:** The results of the mechanical and physical characteristics of the tested cement pastes.

Series	C42			C42MS			C52			C52MS		
*w*/*b*	0.4	0.5	0.6	0.4	0.5	0.6	0.4	0.5	0.6	0.4	0.5	0.6
*f_c(R)_* [MPa]	61.40	43.43	34.17	61.97	46.88	35.47	69.52	49.67	39.10	68.13	50.99	41.01
*f_c(T)_* [MPa]	39.26	23.08	15.90	41.45	25.80	14.70	49.29	30.42	21.13	52.54	41.07	24.40
*f_cf(R)_* [MPa]	5.80	4.35	3.16	5.10	4.63	3.03	5.74	4.39	3.86	6.26	3.94	3.19
*f_cf(T)_* [MPa]	2.97	2.59	1.32	3.26	2.55	1.36	1.53	1.35	1.16	1.94	1.79	1.27
*D_(R)_* [g/cm^3^]	1.692	1.528	1.416	1.669	1.488	1.337	1.750	1.574	1.432	1.710	1.571	1.388
*D_(T)_* [g/cm^3^]	1.513	1.376	1.277	1.489	1.329	1.196	1.549	1.388	1.266	1.511	1.367	1.219

**Table 3:** Values of the coefficients of variation.

Series	C42			C42MS			C52			C52MS		
*w*/*b*	0.4	0.5	0.6	0.4	0.5	0.6	0.4	0.5	0.6	0.4	0.5	0.6
*f_c(R)_* [%]	7.24	4.42	4.19	5.08	4.61	2.97	7.58	5.31	4.79	2.96	4.92	5.06
*f_c(T)_* [%]	4.85	4.15	3.39	3.42	3.40	2.77	5.71	3.67	4.11	7.38	5.59	6.82
*f_cf(R)_* [%]	3.52	8.06	7.44	2.63	6.87	2.41	8.46	4.64	7.19	1.99	5.51	3.94
*f_cf(T)_* [%]	7.98	7.99	2.16	4.62	4.49	7.40	8.57	8.15	5.90	2.76	5.38	5.37
*D_(R)_* [%]	0.25	0.20	0.15	0.29	0.35	0.24	0.33	0.41	0.56	0.10	0.41	0.40
*D_(T)_* [%]	0.22	0.08	0.24	0.33	0.32	0.25	0.34	0.31	0.45	0.28	0.37	0.56

**Table 4:** Equations of curves describing the $\bar{A}\left(\bar{I}\right)$ relationship, together with values of diagnostic statistics (*R_2_*, *S_e_*, *W*) and the correlations coefficients (*ρ*).

Series	Equation	*R* ^2^	*S_e_* [mm^2^]	*W* [%]	$\rho —\bar{A}/\bar{I}$	
C42	y = 53570x^2^ + 808.28x	0.82	33.39	20.82	0.75	C42
C42MS	y = 40368x^2^ + 1762.60x	0.91	11.38	10.03		C42MS
C52	y = 20643x^2^ + 762.54x	0.94	15.28	13.42		C52
C52MS	y = 75147x^2^ − 205.88x	0.91	10.30	12.60		C52MS

**Table 5:** Composition of the cement pastes tested after the thermal interaction, together with a percentage difference in the mass content of individual components, with respect to the C52 sample.

Oxide	Percentage of Oxides (Percentage Difference with Respect to C52) [%]	
	C52	C52MS
Na_2_O	0.79 (−)	0.59 (−25.6)
MgO	1.06 (−)	1.18 (12.0)
Al_2_O_5_	6.58 (−)	4.85 (−26.3)
SiO_2_	22.87 (−)	26.80 (17.2)
SO_3_	3.78 (−)	4.20 (10.9)
K_2_O	0.52 (−)	0.70 (34.4)
CaO	61.30 (−)	59.12 (−3.6)
Fe_2_O_3_	3.12 (−)	2.55 (−18.3)
Na_2_O	0.79 (−)	0.59 (−25.6)
